# Polarized Th2 cells attenuate high-fat-diet induced obesity through the suppression of lipogenesis

**DOI:** 10.1186/s12865-024-00598-z

**Published:** 2024-01-10

**Authors:** Lijun Dong, Jingtao Gao, Lu Yu, Shibo Liu, Yuxin Zhao, Wen Zhang, Yinming Liang, Hui Wang

**Affiliations:** 1 Henan Key Laboratory of Immunology and Targeted Drug, School of Laboratory Medicine, Xinxiang Medical University, Henan Province, Xinxiang, 453003 PR China; 2 Henan Collaborative Innovation Center of Molecular Diagnosis and Laboratory Medicine, Xinxiang Medical University, Xinxiang, PR China; 3 Department of Immunology, Xinjiang Medical University, Urumqi, PR China; 4 Division of Vascular and Interventional Radiology, Department of General Surgery, Nanfang Hospital, Southern Medical University, Guangzhou, 510515 Guangdong China

**Keywords:** Th2, High fat diet, Obesity, Adipocyte, Lipogenesis

## Abstract

Immune cells, such as macrophages, B cells, neutrophils and T cell subsets, have been implicated in the context of obesity. However, the specific role of Th2 cells in adipose tissue function has remained elusive. Eight-week-old male CD3ε^─/─^ mice were randomly divided into two groups (≥ 5 mice per group): one received intravenous injection of Th2 cells isolated from LAT^Y136F^ mice, while the other receiving PBS as a control. Both of groups were subjected to a high-fat diet (HFD). The adoptive transfer of polarized Th2 cells led to a significant reduction in obesity following a HFD. This reduction was accompanied by improvements in hepatic steatosis, glucose intolerance, and insulin resistance. Mechanistically, Th2 cell treatment promoted oxidative phosphorylation of adipocytes, thereby contributing to a reduction of lipid droplet accumulation. These findings suggest that Th2 cell therapy represents a novel approach for treating diet-induced obesity and other diseases involving lipid droplet accumulation disorders.

**Supplementary Information:**

The online version contains supplementary material available at 10.1186/s12865-024-00598-z.

## Introduction

Obesity is a global health concern associated with various related conditions, including type 2 diabetes [1], fatty liver disease [2], atherosclerosis [3], cardiovascular problems, cancer [4] and autoimmune diseases [5]. multiple factors contribute to obesity such as diet, physical activity, genetics, environment, stress, emotion and sleep quality. Notably, high-fat diet has emerged as the primary driver of obesity, especially in Western countries. White adipose tissue plays a pivotal role in regulating energy balance by influencing both food consumption and energy expenditure [6]. White adipocytes, the predominant cell type in white adipose tissue, store dietary energy in a highly concentrated form as triglyceride, mostly in a single large lipid droplet. Unlike the number of adipocytes, which remains relatively constant in adulthood, the size of these cells can increase due to intracellular triglyceride accumulation. The content of lipid droplets within adipocytes is a critical factor in determining obesity within white adipose tissue. Consequently, targeting adipocytes presents a significant strategy to address the obesity epidemic.

In addition, obesity is now recognized as a chronic low-grade inflammatory disease,closely linked to ongoing inflammation within adipose tissue. Typically, in acute inflammation, the initial damaging insult triggers the release of a number of immunomodulatory molecules, including cytokines and chemokines by tissue resident macrophages and mast cells, provoking a rapid recruitment of neutrophils, followed by macrophages and lymphocytes from the circulation to the inflammation site [7]. A recent study has proposed that adipocytes and macrophages utilize intercellular mitochondria transfer as a mechanism of immunometabolic crosstalk, which plays a critical role in regulating metabolic homeostasis. Notably, this mechanism becomes impaired in obesity [8]. Furthermore, dendritic cells [9], mast cells [10], neutrophils [11], eosinophils [12] and T cell subsets (CD8 + T cells [13], Th17 [14], Th1 [15], Tregs [16], NKT [17]) also participate in high-fat diet induced obesity and related metabolic disorders. It is worth mentioning that obesity aggravated the immune histopathological characteristics in an experimental model of eosinophilic esophagitis, which was associated with the reduction of the regulatory profile and the increased influx of inflammatory cells, related to the Th2 profile [18]. The frequency of Th2 cells in WAT is negatively correlated with systemic inflammation and insulin resistance in mice and humans, indicating that Th2 cells have a protective function [19]. In this study, we investigate the impact of polarized Th2 cells on mice subjected to a high-fat diet (HFD). We further show that Th2 cells can alleviate fat accumulation of adipose tissue and steatosis in mice fed on HFD.

## Materials and methods

### Mice and treatments

Male LAT^Y136F^ mutant C57BL/6 J mice and CD3ε knockout C57BL/6 J mice were obtained from Centre d’Immunologie de Marseille-Luminy in France. Mice were maintained under specific pathogen-free (SPF) conditions. All animal experiments in this study were approved by the Welfare and Ethical Committee for Experimental Animal Care of Xinxiang Medical University. These mice were kept under standard laboratory conditions with a controlled temperature (19–22 °C) and a 12-h light/dark cycle. Purified Th2 cells were obtained from mesenteric lymph nodes of LAT^Y136F^ mutant C57BL/6 J mice with using CD5 MicroBeads (130–049-301 Miltenyi) following the standard protocol. Eight-week-old CD3ε^─/─^ male mice were randomly divided into two groups (n ≥ 5): one receiving a 60% HFD with PBS injection and the other receiving a 60% HFD with intravenous injection of Th2 cells isolated from LAT^Y136F^ mice. 2 × 10^6^ purified Th2 cells in 200 μl PBS or 200 μl PBS only were injected into CD3ε knockout C57BL/6 J mice via tail vein respectively. Afterward, the two group of mice were subjected to high-fat diet (D12492i, Research Diets, 60 kcal%) for 16 weeks. At the end of the experimental period, the mice were euthanized with 4% isoflurane inhalation.

### Metabolic and physical activity measurements

Animals were placed individually in chambers for 5 consecutive days at ambient temperature with 12 h light/dark cycles. Animals had free access to food and water. At the end of 16 weeks, the mice were given 2 days of acclimation in metabolic chambers before the trial and then continuously recorded for 96 h. Measurements were taken every 30 min, including food intake, water intake, ambulatory activity (in X and Z axes), and gas exchange (O2 and CO2). All measurements were taken automatically using the SABLE SYSTEMS INTERNATIONAL (USA).

### Glucose and insulin tolerance test

The glucose tolerance test (GTT) and insulin tolerance test (ITT) were conducted in mice after a 6-h fast. The mice were intraperitoneally injected with 2 g/kg of glucose for GTT, and 0.75 U/kg of insulin for ITT. In the studies, the blood glucose was tested at six time points (0, 15, 30, 60, 90 and 120 min).

### RNA isolation and real time RT-PCR

Total RNA was isolated from animal tissues with TRIzol (Invitrogen, Carlsbad, CA). RNA concentration was measured with a spectrophotometer (Thermo Scientific, Ltd, Waltadult mice, MA). The first strand of cDNA was obtained using the PrimeaScript™ II 1st Strand cDNA synthesis kit (Takara, Ltd, Japan) according to the manufacturer’s instruction. Subsequently, SYBR green-based qPCR was performed using a power SYBR greenmaster mix (Applied Biosystems, Foster City, CA) and 7500 Fast Real-Time PCR system (Applied Biosystems, Foster City, CA). The expression was normalized to mouse ribosome 18S rRNA.

### Western blots

EAT and liver were collected from mice, snap frozen in liquid nitrogen and homogenized in RIPA buffer with protease inhibitors. Homogenates were cleared by centrifugation at 10000 rpm for 30 min at 4 °C. Protein lysates (30 μg) were separated on 10% SDS-PAGE gels and transferred onto polyvinyldifluoride membranes (Millipore, Bedford, USA). Membranes were blocked in tris-buffered saline (TBS, pH 7.4) containing 5% nonfat milk at room temperature for 1 h and then incubated with primary antibodies overnight at 4 °C. After washing, the membranes were incubated with HRP-conjugated secondary antibodies at room temperature for 1 h. Detection and analysis were performed using Chemidoc XRS Image system and Image Lab 5.0 software (Biorad). The blots are cut to the appropriate size prior to hybridisation with antibodies. Cropped images were adopted is presented in Supplementary information.

### Cell culture and induction of adipocyte differentiation

3T3-L1 cells were maintained in DMEM supplemented with 10% calf serum and 5% CO2. For adipocyte differentiation, the cells were treated with 10 μg/mL insulin, 1 μM dexamethasone and 0.5 mM 1-methyl-3-isobutylxanthine (IBMX) in DMEM containing 10% (v/v) fetal bovine serum (FBS) and 1% penicillin–streptomycin for 2 days. The medium was then replaced by a medium containing only insulin. After an additional 2 days, this maintenance medium was discarded. The differentiated adipocytes were cultured in DMEM containing 10% FBS.

### Histology

Mice were euthanized at indicated time points and adipose tissue and liver were carefully harvested. Frezon section was applied for oil red staining, and the liver tissue were placed in a sealed box and embedd with OCT (Leica). For hematoxylin and eosin (H&E) staining, tissues were fixed in 4% paraformaldehyde (PFA) overnight at 4℃, followed by dehydration in 70% ethanol (Leica ASP300S). After the dehydration procedure, tissues were embedded in paraffin, sectioned at a thickness of 5 mm, and stained with H&E following the standard protocol. Images were acquired using a LEICA 500 microscope.

### Co-culture of Th2 Cells and 3T3-L1 Preadipocytes

3T3-L1 preadipocyte cells were plated in 24 wells at a density of 1 × 10^5^ cells/well and maintained in DMEM supplemented with 1% penicillin/streptomycin and 10% FBS. To evaluate the effect of Th2 cells on 3T3-L1 induction and differentiation, 2-days-postconfluent cells (day 0) were treated with different numbers of purified Th2 cells (5 × 10^4^ and 1 × 10^5^ Th2 cells) and induction medium I (DMEM contains10% FBS, 1.73 μM insulin, 1 μM dexamethasone, 0.5 mM 3 -isobutyl-1-methylzanthine, 0.2 μM indomethaein and 1 μM pioglitazone) every 2 days until day4, while control group was treated without Th2 cells. The medium was replaced with induction medium II (DMEM contains10% FBS and 1.73 μM insulin) every 2 days until day 8.

### Plasma cytokines measurement

Whole blood samples were centrifuged (5,000 g, 10 min at 4 °C), and plasma fraction was stored at –80 °C. Plasma levels of cytokines were assessed with a Magnetic Luminex assay kit according to the manufacturer's instructions.

### Cholesterol quantification

Total cholesterol, HDL and LDL quantification was done using the Cholesterol/Cholesteryl Ester Assay Kit and (Abcam, ab65359 and ab65390). Triglyceride of mice plasma was detected by Triglyceride (TG) Colorimetric Assay Kit (Thermo Fisher, EEA028). The plasma fraction of mice was analyzed according to the manufacturer’s instructions.

### Flow cytometric analysis

Mice's epididymal adipose tissues were taken, and they were digested for an hour at 37 °C with collagenase type I at a concentration of 1 mg/mL. After centrifuging the digested tissues through a 100 μm filter, the cells were recovered. Red blood cells were eliminated by resuspending the stromal vascular cells in red blood cell lysis solution for five minutes. An FcR-specific blocking monoclonal antibody was used to pre-incubate single-cell suspensions, and then they were washed before staining. The cells were stained with APC-conjugated anti-CD45 (0.1 μg/mL), BV510-conjugated anti-CD5 (0.2 μg/mL), PE-Cy7-conjugated anti-CD4 (0.2 μg/mL), and APC-Cy7- conjugated anti-CD8 (0.5 μg/mL) (using 1000 dilutions in the PBS) in the dark for 30 min followed by PBS washing and resuspended in PBS supplemented with 5% fetal calf serum. All of the fluorescent antibodies were purchased from BioLegend, Inc. (CA, USA). The date were recorded in a FACSCanto II (BD Biosciences), and analyzed using the FlowJo software.

### Statistical analysis

All results were expressed as mean ± SEM (standard error of the mean). Statistical significance between two groups was evaluated using the Student's t-test. For morphometric analyses, quantification of micrographs was independently reviewed by two observers and the average of their scores was used for each micrograph. Quantification was performed on images after appropriate thresholding using the Image J software (NIH Image).

## Results

### Polarized Th2 cells protect against HFD induced obesity of mice

**Fig. 1:**
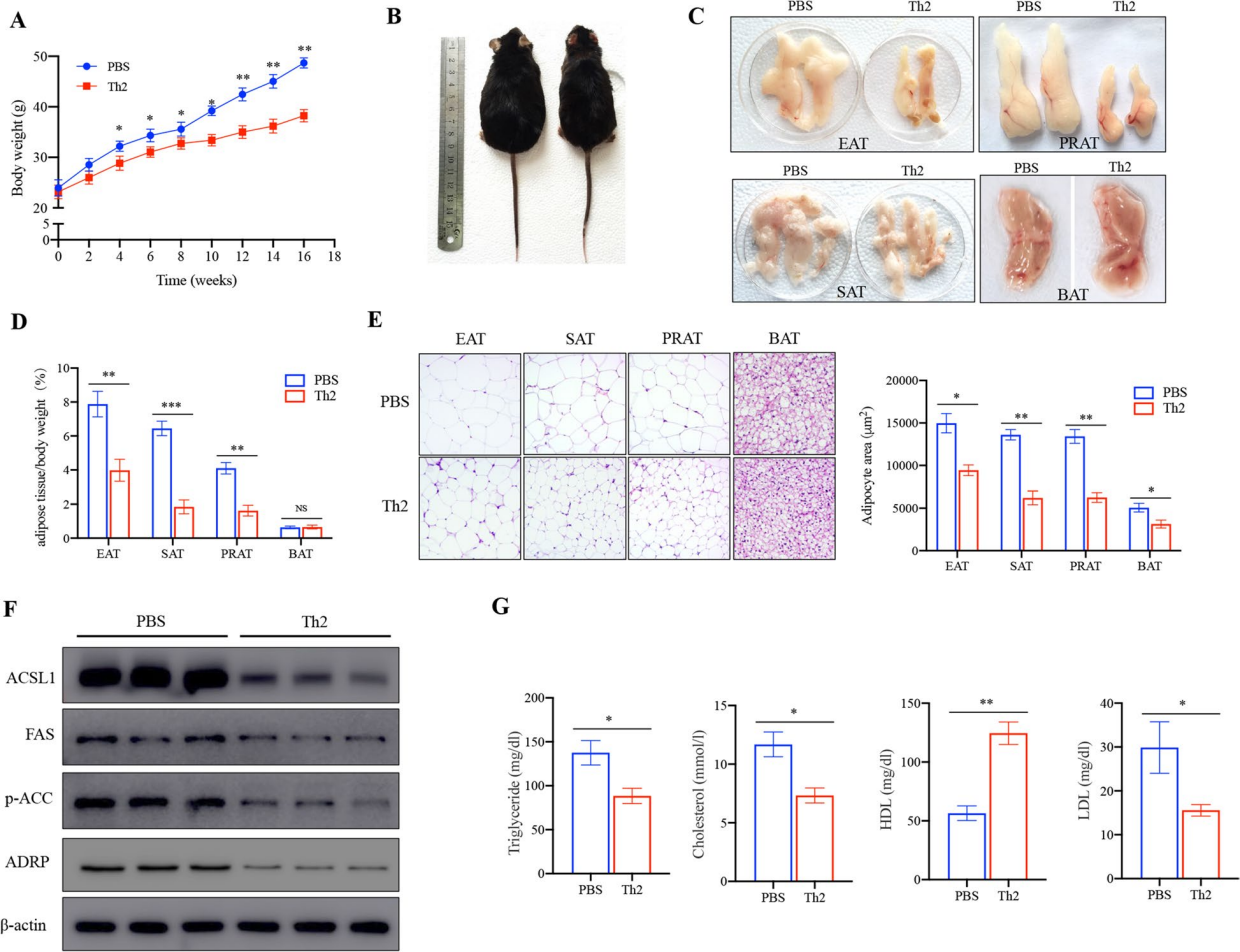
Th2 cells reduce HFD-induced obesity of mice. Eight-week-old CD3ε^─/─^ male mice were intravenous injection of Th2 cells isolated from LAT^Y136F^ mice or PBS in the presence of High-fat diet (HFD) for 16 weeks respectively. **A** The body weight changes during the experiments were monitored. (*n* = 5). **B** Photographs of representative mice fed on HFD. Representative appearance of epididymal adipose tissue (EAT), subcutaneous adipose tissue (SAT), perirenal adipose tissue (PRAT), brown adipose tissue (BAT) (**C**) and ratio of tissue weight to body weight (**D**). **E** Representative H&E staining of EAT, SAT, PRAT and BAT. Scale bars = 50 µm. **F** The protein expression of long-chain acyl-CoA synthetase (ACSL), FAS (fatty acid synthase), Acetyl-CoA Carboxylase (ACC) and Adipose differentiation-related protein (ADRP) was determined by western blot analysis. **G** The serum concentrations of triglyceride (TG), cholesterol (TC), high-density lipoprotein (HDL) and low-density lipoprotein (LDL) were evaluated at the indicated time points. *P < 0.05, **P < 0.01, NS, not significant. Data from one representative experiment of three independent experiments are presented. Cropped images were adopted and full-length blots/gels are presented in Supplementary information

To determine the function of Th2 cells in obesity, we initially evaluated whether lipid accumulation in adipose tissue could be altered by Th2 cells adoptive transfer under HFD condition. Mice that transfered with Th2 cells showed resistance to weight gain after 16 weeks HFD (Fig. 1A), with a noticeably leaner appearance compared to the control group (Fig. 1B). Furthermore, the epididymal adipose tissue (EAT), subcutaneous adipose tissue (SAT), perirenal adipose tissue (PRAT) and brown adipose tissue (BAT) were markedly reduced in size in the recipient mice, consistent with a notable decrease in the size of adipocytes (Fig. 1C, D, E). Western blot analysis further confirmed the reduced protein levels of ACSL (long-chain acyl-CoA synthetase), FAS (fatty acid synthase), Acetyl-CoA Carboxylase (ACC) and lipid droplets marker perilipin-2 in Th2 cells transfer group compared to the control group (Fig. 1F). Additionally, the biochemical analysis revealed that Th2 cells also reduced serum triglyceride (TG), cholesterol (TC) and high-density lipoprotein (HDL) concentrations while increasing serum low-density lipoprotein (LDL) of CD3ε^─/─^ mice fed the HFD (Fig. 1G).

### Th2 cells prevent HFD-induced hepatic steatosis

**Fig. 2:**
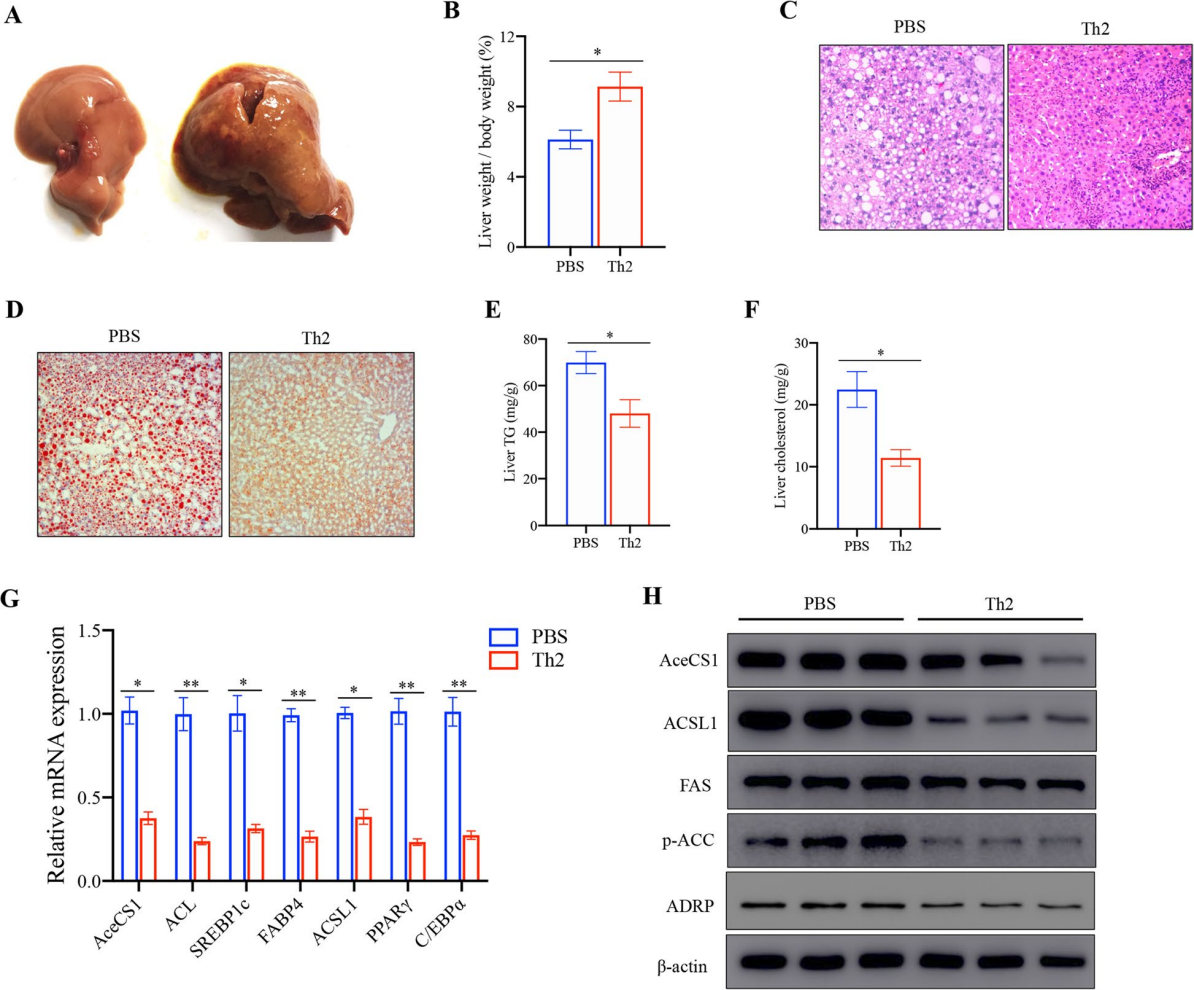
Th2 cells relieve fatty liver. Livers were isolated on the last day of the experiment (*n* = 5 per group). **A** A representative photograph of liver from each group is provided, and (**B**) the liver weight was recorded. **C** The histological analysis of mouse liver was performed by hematoxylin and eosin (H&E), (**D**) and oil red staining. Scale bar = 100 μm. **E** Hepatic TG level. **F** Hepatic cholesterol level. **G** The mRNA levels of acetyl-CoA synthetase (AceCS1), ATP-Citrate Lyase (ACL), sterol regulatory element binding protein 1 (SREBP1c), fatty acid-binding protein 4 (FABP4), long-chain acyl-CoA synthetase 1 (ACSL1), peroxisome proliferator-activated receptor γ (PPARγ) and CCAAT/enhancer-binding proteins (C/EBP) in livers were examined by qRT-PCR analysis. The related protein expression was examined by western blot analysis(H). Cropped images were adopted and full-length blots/gels are presented in Supplementary information

As dietary habits become increasingly Westernized, the global prevalence of non-alcoholic fatty liver disease (NAFLD) has risen to approximately 25% of the world's population [19]. To determine the role of Th2 cells in fatty liver, representative photographs of liver (Fig. 2A) and liver index (Fig. 2B) showed that increased liver weight in Th2 cells-transferred mice compared to control mice. However, adoptive transfer of Th2 cells substantially attenuated the lipid accumulation of liver upon HFD as evidenced by H&E (Fig. 2C) and oil red staining (Fig. 2D). Consistently, Th2 cells transfer also result in reduced levels of triglycerides (Fig. 2E) and cholesterol (Fig. 2F) in the liver. Both mRNA and protein expression of lipid synthesis decreased in the liver of recipient mice on an HFD (Fig. 2G, H).

### Th2 cells reverse HFD-induced metabolic imbalance

**Fig. 3:**
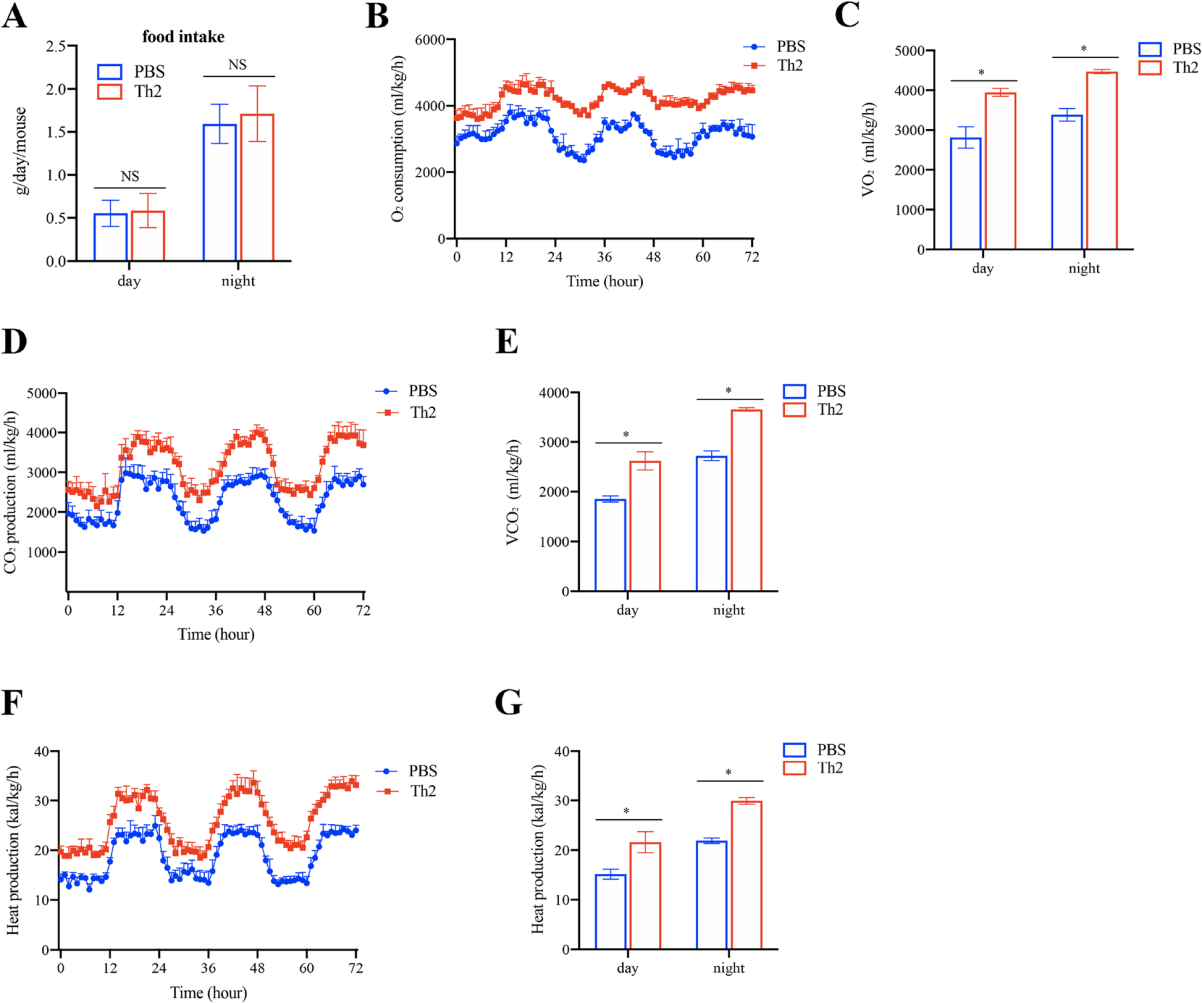
Th2 cells improve metabolic parameter. Eight-week-old CD3ε^─/─^ male mice were intravenous injection of Th2 cells isolated from LAT^Y136F^ mice or PBS in the presence of High-fat diet (60%) for 16 weeks respectively (n = 5 per group). **A** Food intake, (**B**, **C**) O_2_ consumption, (**D**, **E**) CO_2_ production, and (F, G) heat production during both the light and dark cycles determined by CLAMS

To explore the potential role of Th2 cells in the regulation of food intake and whole-body energy homeostasis during HFD, mice were monitored for energy expenditure and food intake. After 16 weeks of exposure to the HFD, there were no significant differences in food intake observed, which was corroborated through in-depth analysis using an automated food intake monitoring system (Fig. 3A). In accordance with the increased body weight gain and elevated body fat composition, a significant impairment in whole-body energy expenditure including O2 consumption and CO2 production were observed in the CD3ε^─/─^ mice but not in the Th2 cells-transferred CD3ε^─/─^ mice (Fig. 3B-E). Moreover, Th2 cell transfer also led to an increase in heat production under HFD conditions (Fig. 3F, G). These results suggest that Th2 cells transfer drastically restore whole-body energy expenditure during HFD conditions.

### Th2 cells improve glucose homeostasis and insulin sensitivity

**Fig. 4:**
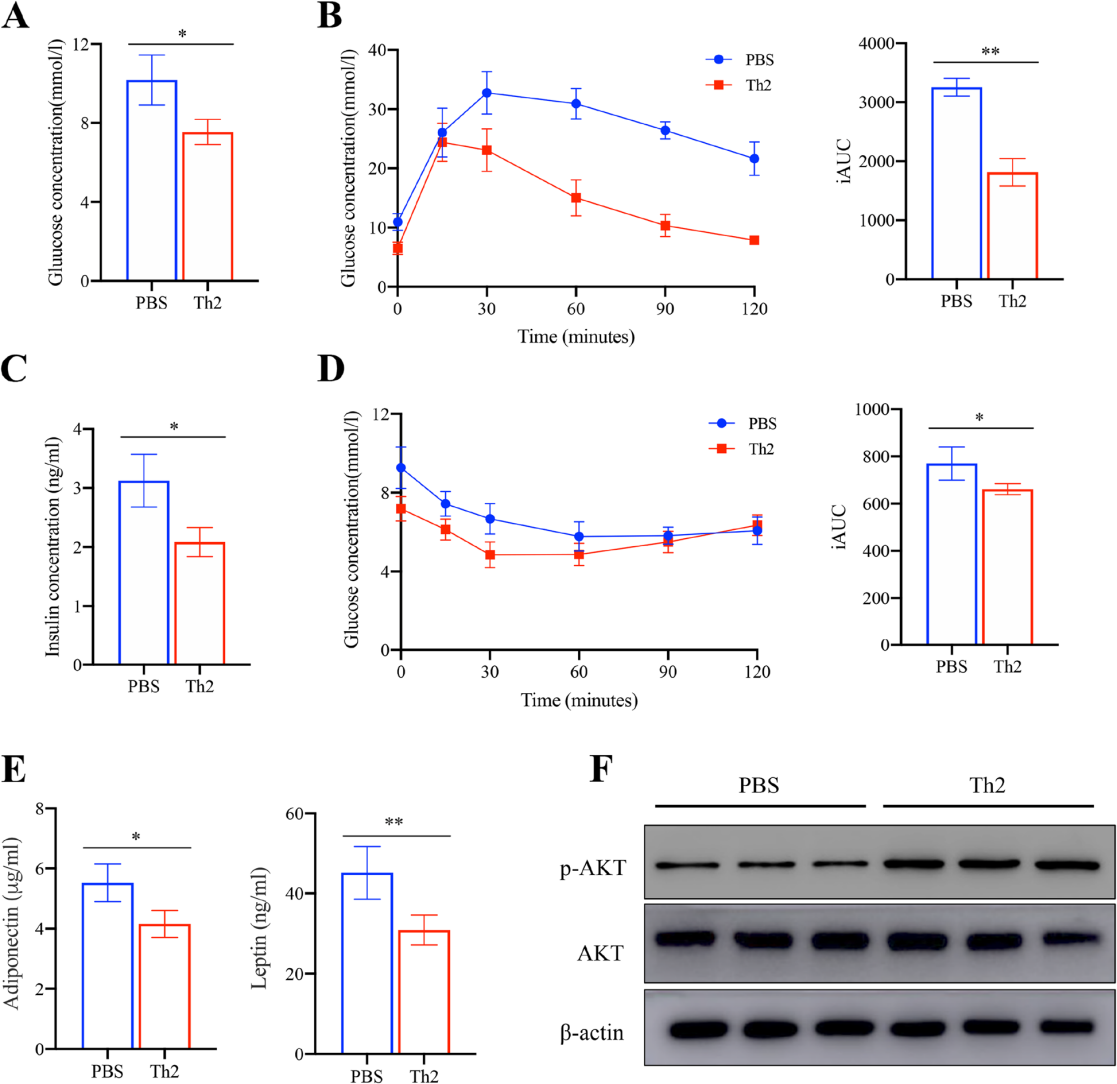
Th2 cells remit glucose intolerance and insulin resistance. **A** Fasting plasma glucose levels, (**B**) insulin levels, (**C**) intraperitoneal glucose tolerance test plasma glucose concentrations, (**D**) insulin tolerance test, (**E**) plasma adiponectin and leptin were measured at 24 weeks of age. Areas under the curve were compared. Immunoblots of phospho-Ser473 Akt (p-Akt), and Akt in the (**F**) the levels of p-Akt in epididymal adipose tissue (EAT) were normalized to Akt. Values shown are mean ± SD (*n* = 5). *P < 0.05 and **P < 0.01. Cropped images were adopted and full-length blots/gels are presented in Supplementary information

To explore the effects of Th2 cells in metabolic related parameters, fasting glucose and insulin levels in mice plasma after 6 h fasting. The transfer of Th2 cells resulted in a significantly decrease in both glucose and insulin concentration (Fig. 4A, C). Furthermore, mice that received Th2 cells also showed improved performance in both the glucose tolerance test (GTT) and insulin tolerance test (ITT) (Fig. 4B, D). Additionally, adiponectin and leptin level of Th2 cells-transferred mice were lower compared to the control mice upon HFD treatment (Fig. 4E). Western blot analysis further confirmed the increased protein level of phospho-AKT in Th2 cells transfer group compared to the control group (Fig. 4F). Taken together, these data indicate that Th2 cells transfer improves glucose tolerance and insulin sensitivity.

### Th2 treatment CD3KO mice show Th2 type inflammation

**Fig. 5:**
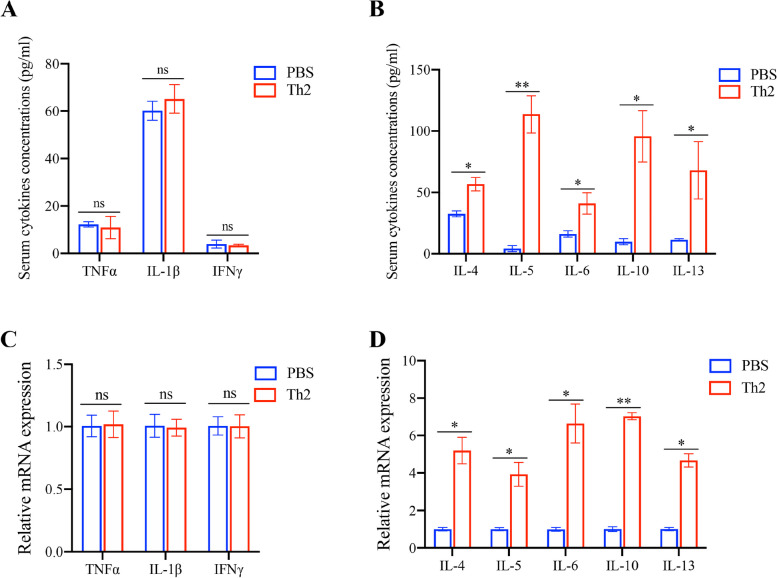
Th2 cells cause Th2 type inflammation. **A**, **B** The serum level of TNF-α, IL-1β, IFN-γ, IL-4, IL-5, IL-6, IL-10 and IL-13 were examined by Luminex High Performance Assay. **C** The mRNA levels of pro-inflammatory cytokine (TNF-α, IL-1β and IFN-γ) and (**D**) Th2-type cytokines (IL-4, IL-5, IL-6, IL-10, IL-13) in eWAT were determined by qRT-PCR analysis

Given the close relationship between inflammation and glucose intolerance as well as insulin resistance, we explored systemic and local inflammation in mice with or without Th2 cells transfer. Pro-inflammatory cytokines such as tumor necrosis factor-α (TNF-α), interleukin 1-β (IL-1β) and interferon-γ (IFN-γ) have no difference in serum and EAT of mice that received Th2 cells compared to the control mice (Fig. 5A, C). We also examined and compared the levels in Th2-type cytokines between CD3ε^─/─^ mice and Th2 cells-transferred CD3ε^─/─^ mice. The results showed that decreased metabolic disorder in recipient mice was accompanied by a significant elevation of Th2-type cytokines, including IL-4, IL-5, IL-6, IL-10, and IL-13 (Fig. 5B, D). In summary, Th2 cells contribute to the heightened expression of Th2-type cytokines in the serum and EAT of mice, which could be a pivotal factor in alleviating metabolic disorders in mice exposed to a high-fat diet.

### Th2 cells inhibit adipocyte differentiation

**Fig. 6:**
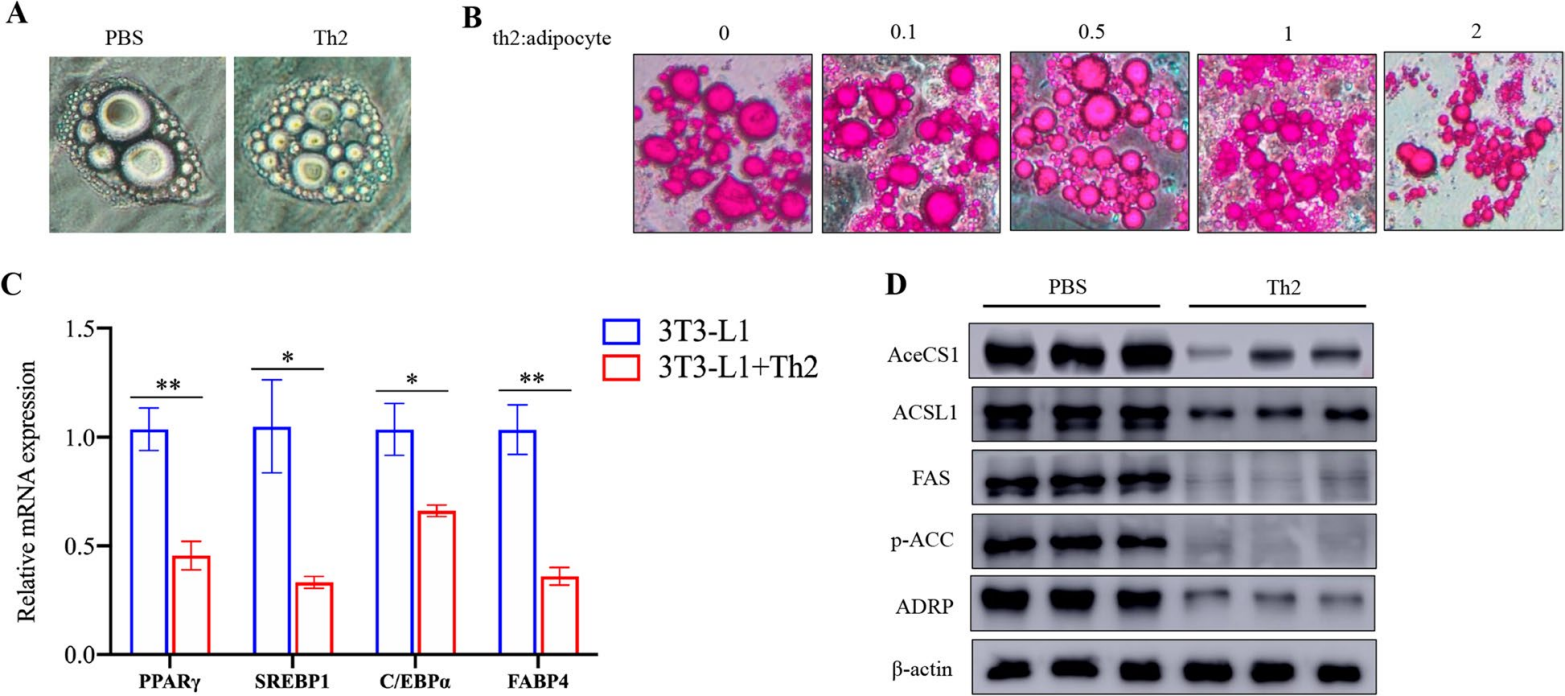
Th2 cells inhibit adipocyte differentiation. 3T3-L1 preadipocyte cells were induced into adipocytes firstly and then co-cultured with different number of Th2 cells for 24 h. **A** Morphology of fat cells were recorded in bright field. **B** Adipocytes differentiation was determined by staining with oil red with different numbers of Th2 cells. Scale bar = 50 μm. **C** The mRNA levels of peroxisome proliferator-activated receptor γ (PPARγ), sterol regulatory element binding protein 1 (SREBP1c), CCAAT/enhancer-binding proteins (C/EBP) and fatty acid-binding protein 4 (FABP4) in adipocytes were determined by qRT-PCR analysis. **D** The protein level of acetyl-CoA synthetase (AceCS1), long-chain acyl-CoA synthetase (ACSL), FAS (fatty acid synthase), phosphorylated Acetyl-CoA Carboxylase (ACC) and Adipose differentiation-related protein (ADRP) was evaluated by immunoblotting analysis. Cropped images were adopted and full-length blots/gels are presented in Supplementary information

The excessive accumulation of lipid droplets in adipocytes is a direct contributor to obesity, Therefore, there is a hypothesis that Th2 cells may play a role in adipocyte differentiation. Our findings support that Th2 cells can indeed inhibit adipocyte differentiation (Fig. 6A, B). The results obtained through qRT-PCR and western blot analysis revealed that, after 7 days of adipogenic differentiation, the expression levels of transcription factors associated with adipocyte differentiation were significantly lower in the Th2 cell treatment group (Fig. 6C, D).

### Th2 cells reduce the accumulation of lipid droplets in adipocyte through mitochondrial oxphos

**Fig. 7:**
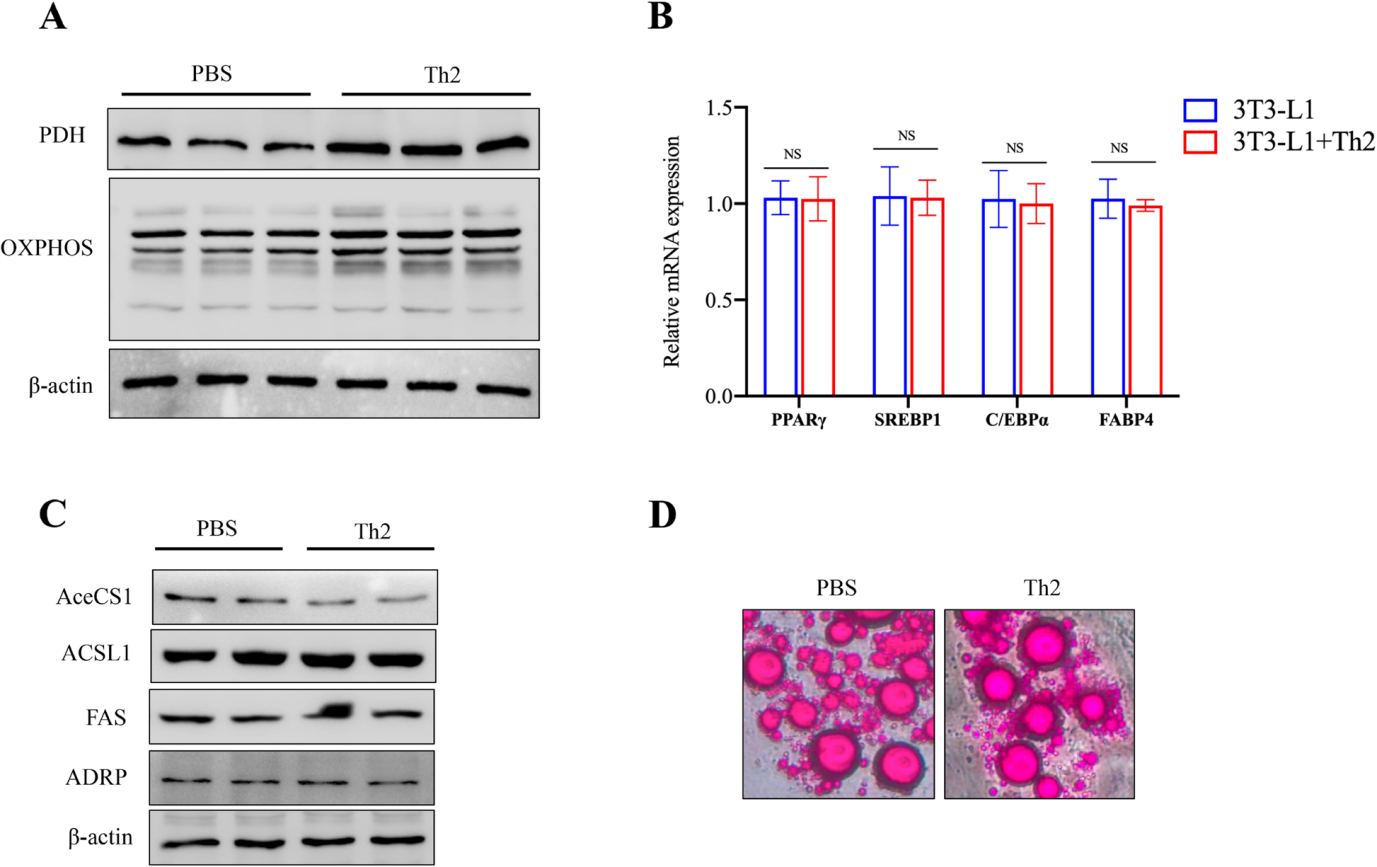
Th2 cells inhibit adipocyte differentiation through oxphos. **A** 3T3-L1 preadipocyte cells were induced into adipocytes firstly and then co-cultured with different number of Th2 cells for 24 h, then the protein levels of PDH and OXPHOS were detected by immunoblotting. **B**-**D** 3T3-L1 preadipocyte cells were induced into adipocytes firstly and then co-cultured with different number of Th2 cells with rotenone for 24 h. **B** The mRNA levels of peroxisome proliferator-activated receptor γ (PPARγ), sterol regulatory element binding protein 1 (SREBP1c), CCAAT/enhancer-binding proteins (C/EBP) and fatty acid-binding protein 4 (FABP4) in adipocytes were determined by qRT-PCR analysis. **C** The protein level of acetyl-CoA synthetase (AceCS1), long-chain acyl-CoA synthetase (ACSL), FAS (fatty acid synthase), and Adipose differentiation-related protein (ADRP) was evaluated by immunoblotting analysis. **D** Adipocytes were determined by staining with oil red. Scale bar = 50 μm. Abbreviations: high-fat-diet (HFD), epididymal adipose tissue (EAT), subcutaneous adipose tissue (SAT), perirenal adipose tissue (PRAT), brown adipose tissue (BAT), long-chain acyl-CoA synthetase (ACSL), FAS (fatty acid synthase), Acetyl-CoA Carboxylase (ACC), triglyceride (TG), cholesterol (TC), high-density lipoprotein (HDL), low-density lipoprotein (LDL), hematoxylin–eosin staining ( H&E staining), acetyl-CoA synthetase (AceCS1), ATP-Citrate Lyase (ACL), sterol regulatory element binding protein 1 (SREBP1c), fatty acid-binding protein 4 (FABP4), peroxisome proliferator-activated receptor γ (PPARγ), CCAAT/enhancer-binding proteins (C/EBP), intraperitoneal glucose tolerance (GTT), insulin tolerance test (ITT)

Given that adipocyte metabolism plays a crucial role in lipid droplet accumulation, we tested the effect of Th2 cells on the level of adipocyte oxidative phosphorylation (oxphos). The results revealed that Th2 cells treatment enhance the expression of mitochondrial aerobic respiration related enzymes (oxphos and PDH) (Fig. 7A). To further validate the role of oxphos in Th2 cells-mediated protection against obesity, adipocytes were pretreated with a specific oxphos inhibitor, rotenone, before Th2 cells treatment. Following rotenone treatment, Th2 cells no longer had affect on lipid droplet accumulation in adipocytes (Fig. 7B, C, D).

## Discussion

T cells, particularly Th2 cells, play a pivotal role in the chronic inflammation observed in the adipose tissue of obese mice. In this study, we present compelling evidence showing a decrease in the frequency of Th2 cells within adipose tissue under high-fat diet (HFD) conditions. Notably, our findings uncover a favorable impact of Th2 cells in the prevention of obesity, primarily through their modulation of lipolysis. We demonstrate that Th2 cells actively enhance mitochondrial energy metabolism in adipocytes by inhibiting fat synthesis while simultaneously elevating oxidative phosphorylation levels.

The lipid accumulation of adipocytes plays crucial roles in the homeostasis of adipose tissue. Dysregulated lipid formation of adipose tissue has been implicated in the development of obesity. Our study shows that Th2 cells protect against obesity and nonalcoholic fatty liver induced by HFD. Indeed, Cytokines secreted by Th2 cells have been linked to the HFD induced obesity as well as the chronic inflammation of adipose tissue. Furthermore, our data show that upregulation of glucose tolerance and insulin sensitivity in the Th2 cells-transferred mice is involved in the AKT pathway.

Obesity is characterized by a chronic, low-grade inflammation. This inflammation involves various immune cells, including macrophages, neutrophils, B cells, and T cells, within adipose tissue. Notably, macrophages comprise up to 40% of all adipose tissue (AT) cells in obese mice, compared to just 10% in lean mice. Our data also suggest that the increased accumulation of macrophages in obese adipose tissue results from the influx of bone marrow-derived precursors into the adipose tissue, where they subsequently differentiate into mature F4/80-expressing macrophages [20]. In addition, Brotfain et al. validated that superoxide production from unstimulated or stimulated neutrophils, along with neutrophil migration, was elevated in obese subjects, while all other neutrophil functions remained normal. This indicates the enhanced capability of neutrophils in combating infections in obese individuals [21]. B cells are also critical regulators of inflammation in T2D due to their direct ability to promote pro-inflammatory T-cell function and secrete pro-inflammatory cytokines [22]. Moreover, T cell subsets including CD8 + T cells (CD8 + effector T cells contribute to macrophage recruitment and adipose tissue inflammation in obesity), Th1 cells [23], and Tregs [24] have been reported to play an important role in diet-induced obesity inflammation. Here, we observed the Th2 cells-transferred CD3ε^−/−^ mice exhibited reduced fat accumulation accompanied by improvement of insulin sensitivity and glucose tolerance compared with CD3ε^−/−^ counterparts, indicating that Th2 cells limit the pathogenesis and progression of obesity. It is widely known that Th2 cells orchestrate protective type 2 immune responses, such as those that target helminths and facilitate tissue repair, but also contribute to chronic inflammatory diseases, such as asthma and allergy. Meanwhile, Th2 cells also perform a regulatory role in the immune system. Therefore, we postulated that Th2 cells might be critical not only in immune regulation but also in metabolic diseases.

The effect of Th2 cells on the protection of obesity could also be manifested through regulating lipid accumulation of adipocytes. As our data suggested, Th2 cells have potent activity in globally decreasing the expression of specific adipocyte markers, as well as the transcription factor PPAR-γ, SREBP1, C/EBPα, and FABP4. It is noteworthy to mention that adipocyte differentiation contributes to the progressive metabolic disorders in patients with obesity. Herein, we provided the evidence for the first time that Th2 cells could inhibit adipocyte differentiation, which strongly expanded our understanding of the pathogenesis of obesity. Dysfunction of mitochondria results in detrimental effects on adipocyte differentiation, lipid metabolism, insulin sensitivity, oxidative capacity, and thermogenesis, which consequently lead to metabolic diseases [25]. To the best of our knowledge, our work reports for the first time that Th2 cells effectively regulate mitochondrial respiration in adipocytes.

## Conclusion

In conclusion, our study conclusively illustrates that Th2 cells effectively mitigate obesity, fatty liver, and metabolic dysfunction induced by a high-fat diet through the reduction of lipid accumulation in adipose tissue. Our mechanistic investigations further unveil that Th2 cells achieve this by inhibiting adipocyte differentiation, a process driven by the enhancement of mitochondrial-mediated aerobic respiration in adipocytes.

### Supplementary Information

**Additional file 1.**

## Data Availability

The data and materials supporting the conclusions of this manuscript will be made available by the authors, without undue reservation, to any qualified researcher.
