# Supplementary material for: Polarized Th2 cells attenuate high-fat-diet induced obesity through the suppression of lipogenesis
Source: BMC Immunol. 2024 Jan 10;25:4. doi: 10.1186/s12865-024-00598-z (PMC10777604; doi:10.1186/s12865-024-00598-z)
Supplement: Supplementary file 1 — Additional file 1. [file 12865_2024_598_MOESM1_ESM.docx]

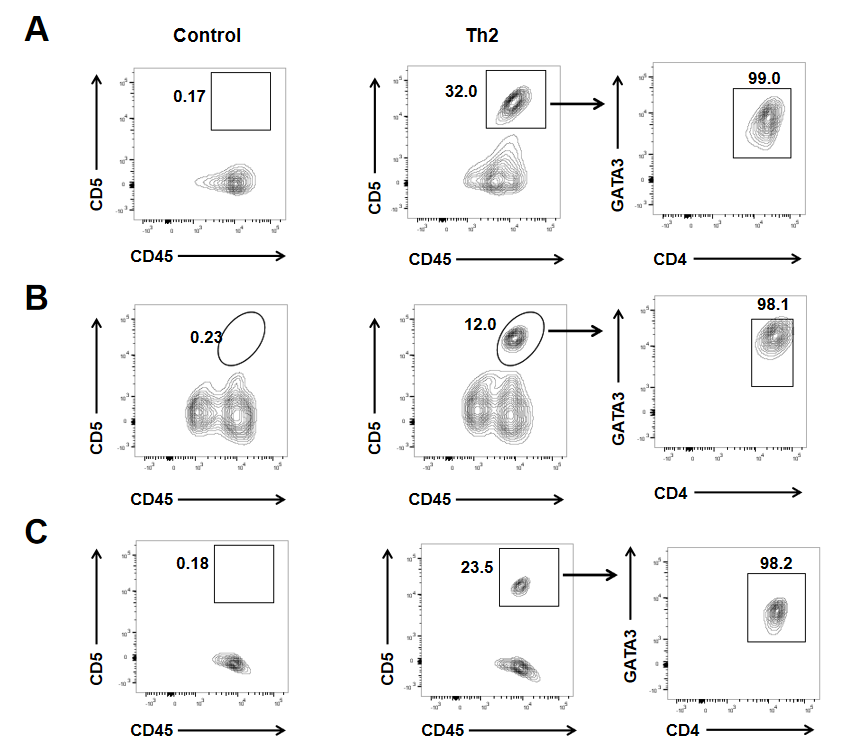


**Supplementary Figure 1: Th2 cells detection in Th2 cells-transferred CD3ε−/− mice**

After 16 weeks of high-fat diet (HFD) exposure, we conducted an investigation into the survival conditions of polarized Th2 cells in different tissues. The results revealed that Th2 cells exhibited robust survival rates in three distinct environments: (A) peripheral blood, (B) adipose tissue, and (C) liver.

a. figure1-ACSL1 b. figure1-FAS


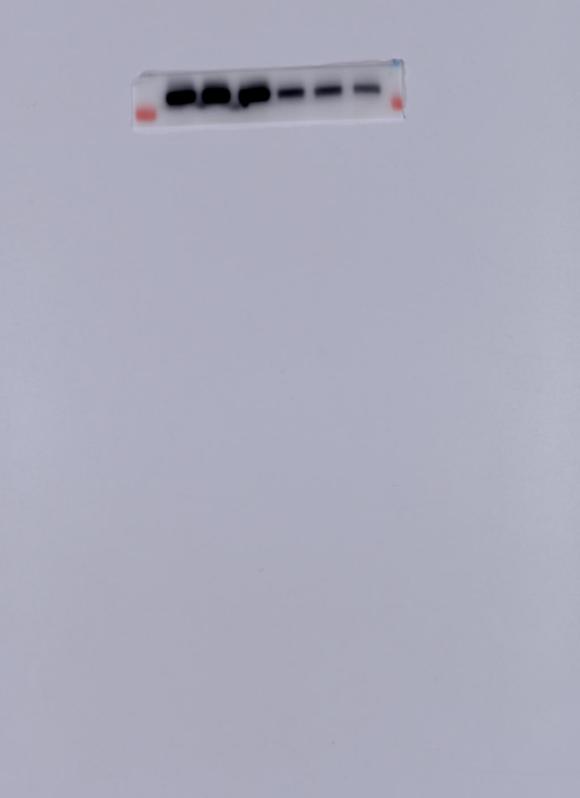

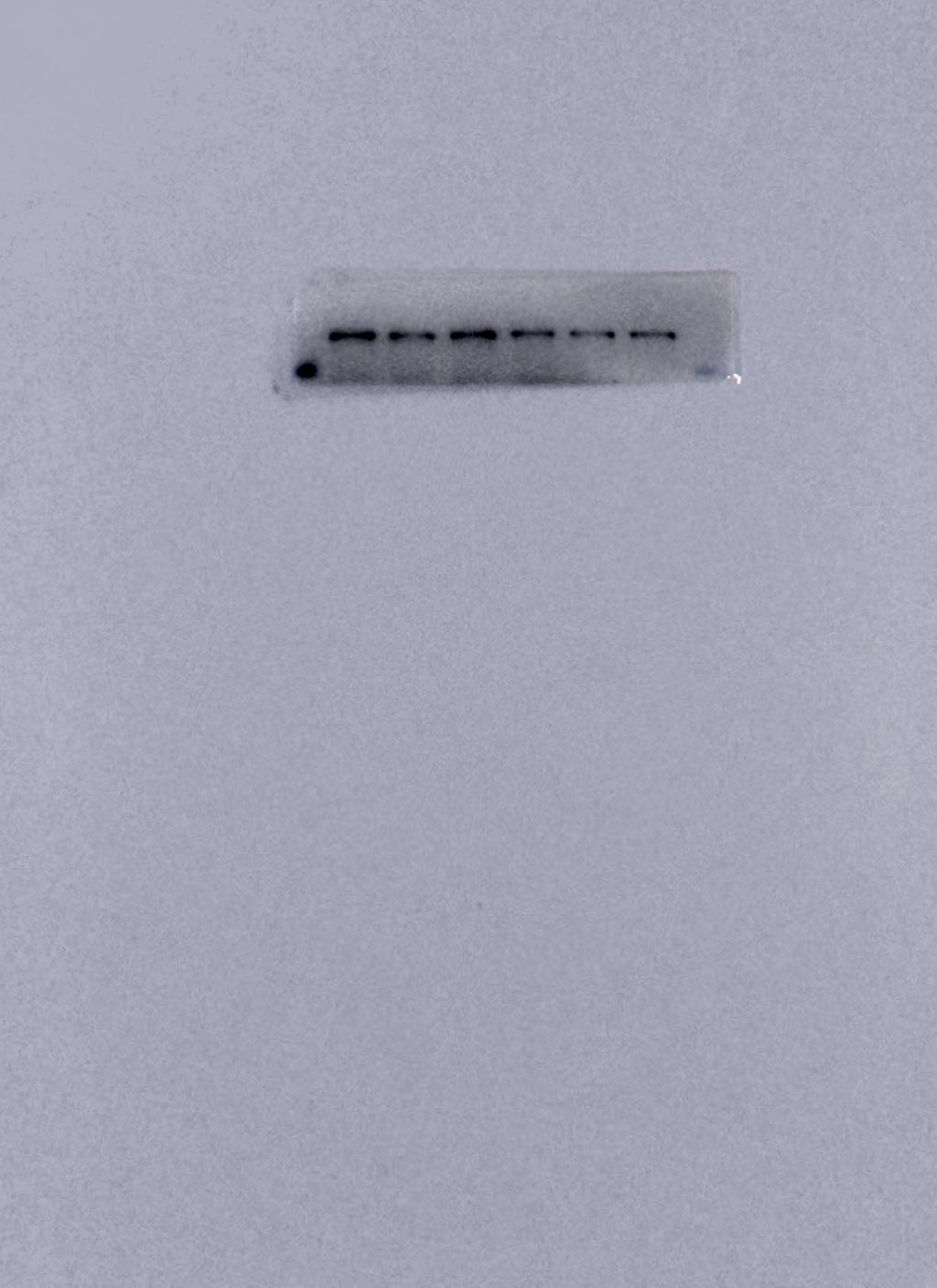


FAS 273 kDa

ACSL1 78 kDa

c. figure1-p-ACC d. figure1- ADRP


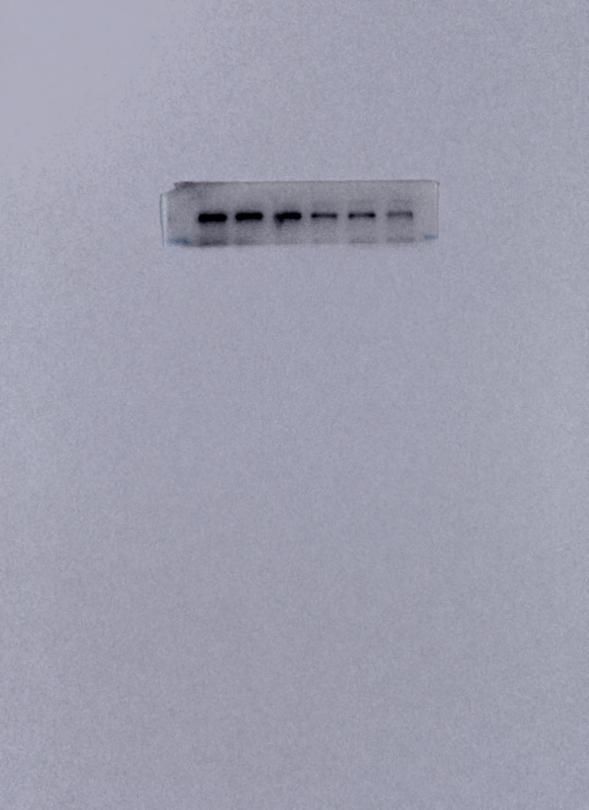

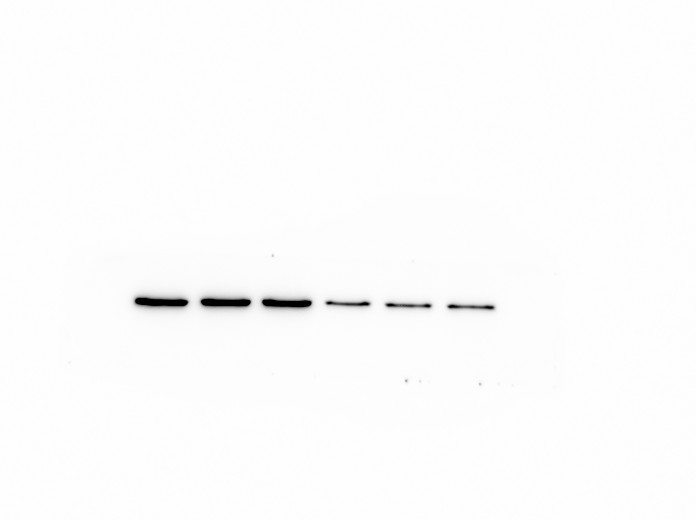


ADRP 52 kDa

p-ACC 280 kDa

d. figure1- ADRP


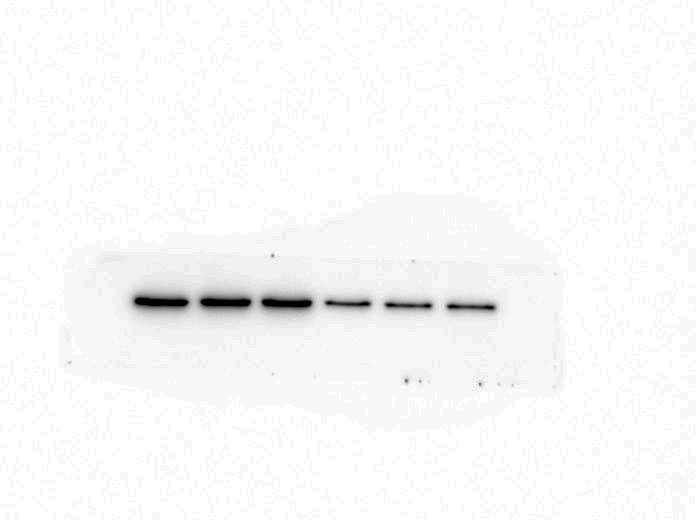


ADRP 52 kDa

e. figure1-β-actin f. figure2-AceCS1


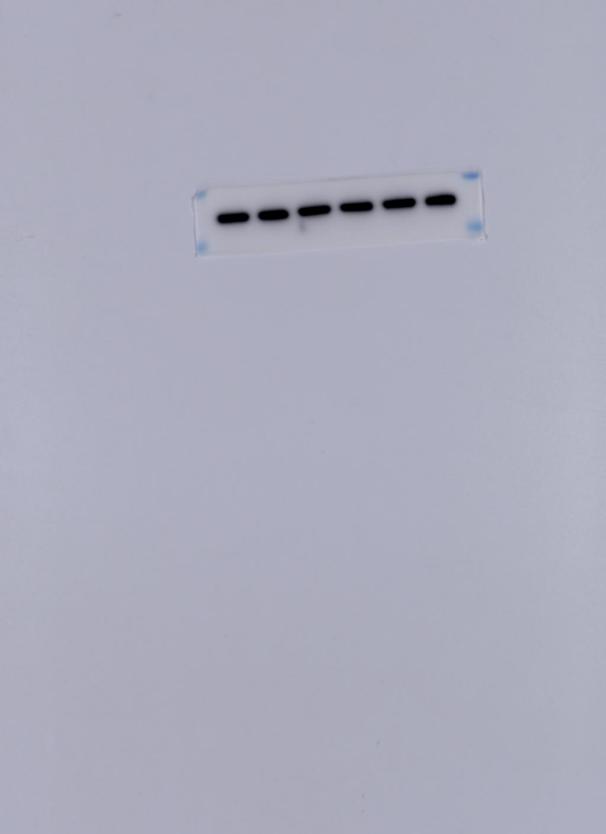

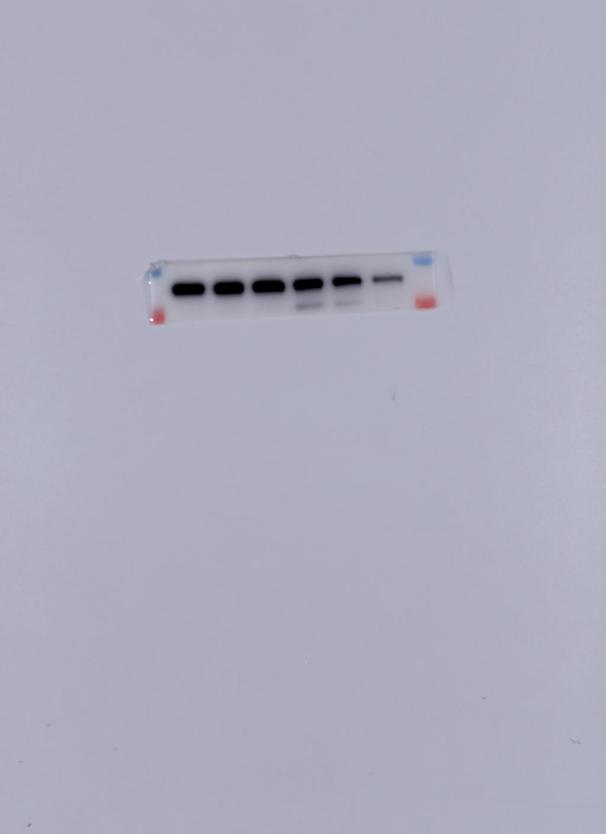


AceCS1 78 kDa

β-actin 45 kDa

g. figure2-ACSL1 h. figure2-FAS


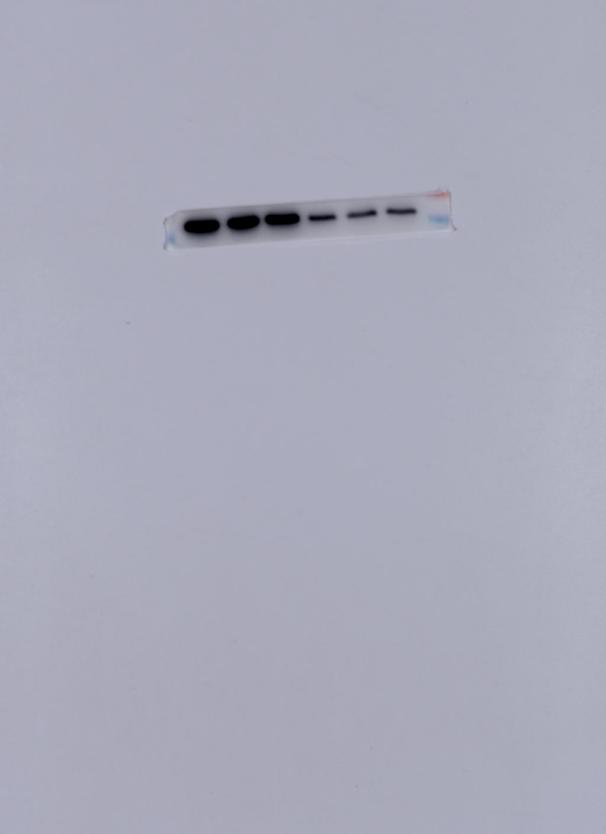

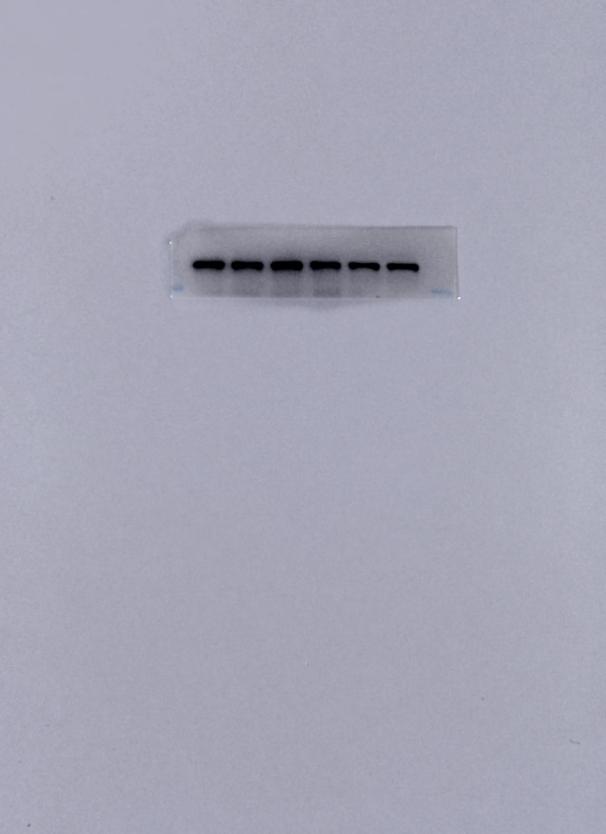


FAS 273 kDa

ACSL1 78 kDa

i. figure2-p-ACC j. figure2- ADRP


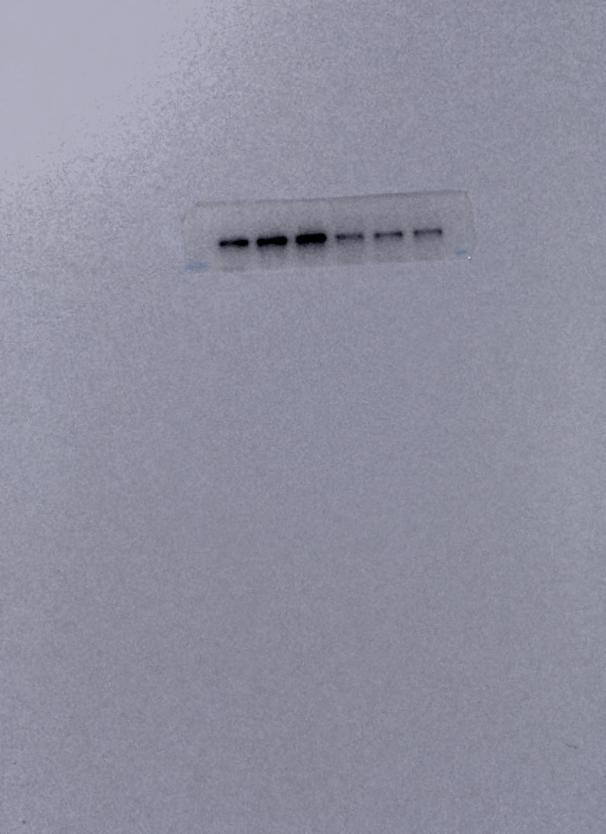

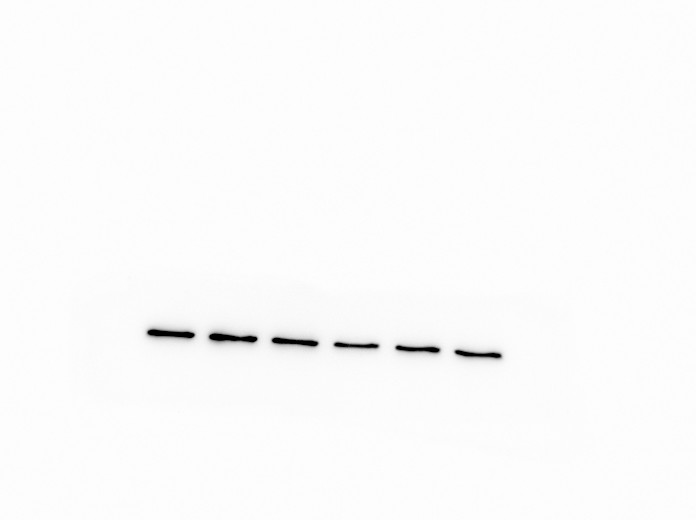


ADRP 52 kDa

p-ACC 280 kDa

j. figure2- ADRP


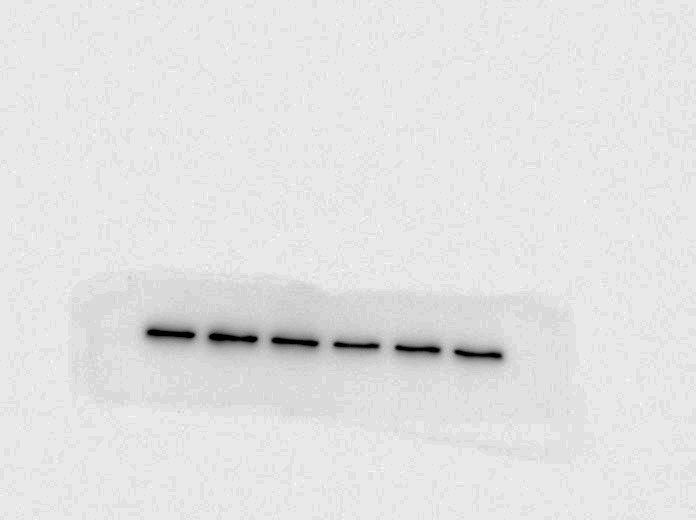


ADRP 52 kDa

k. figure2-β-actin l. figure4-p-AKT


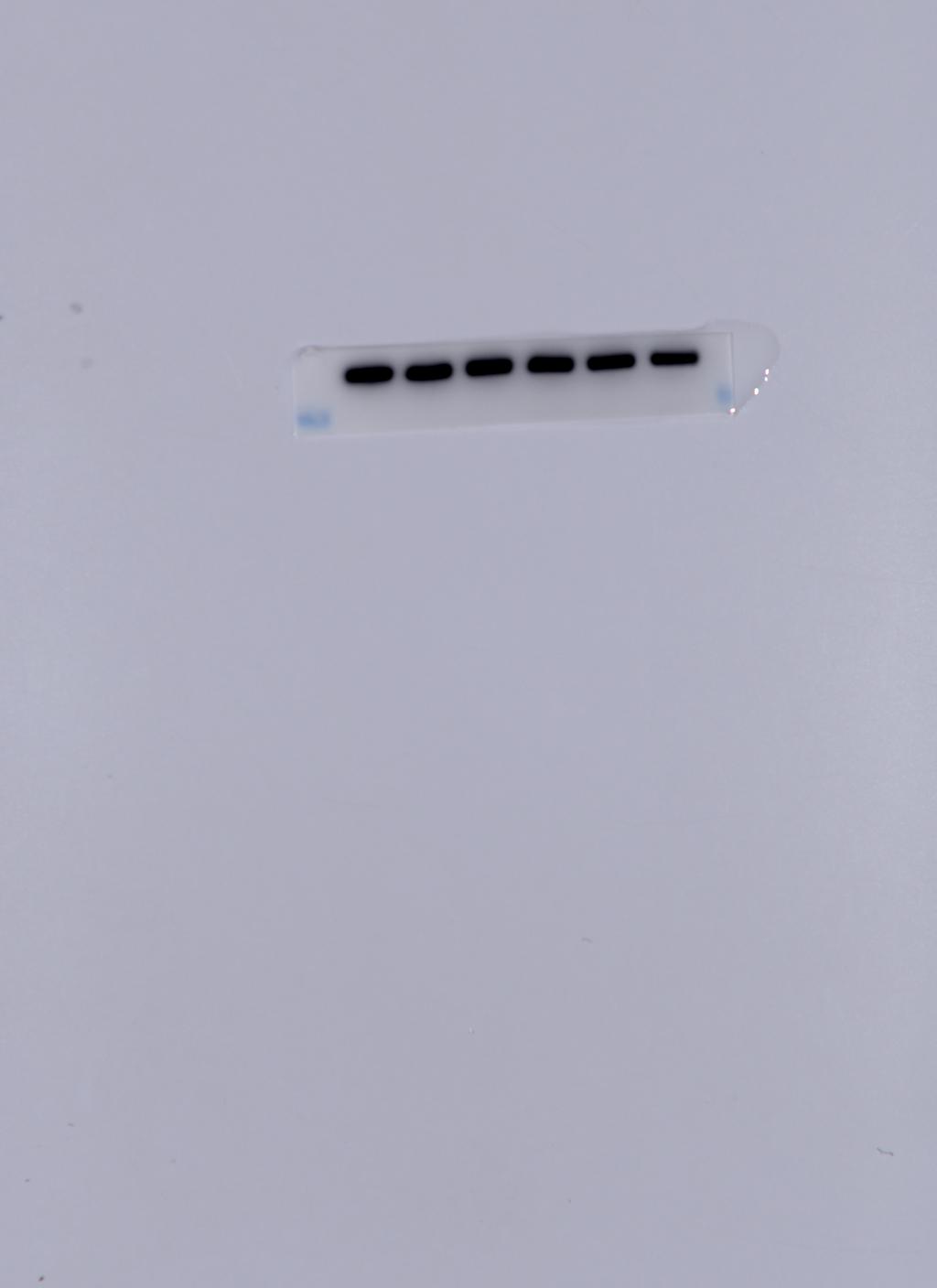

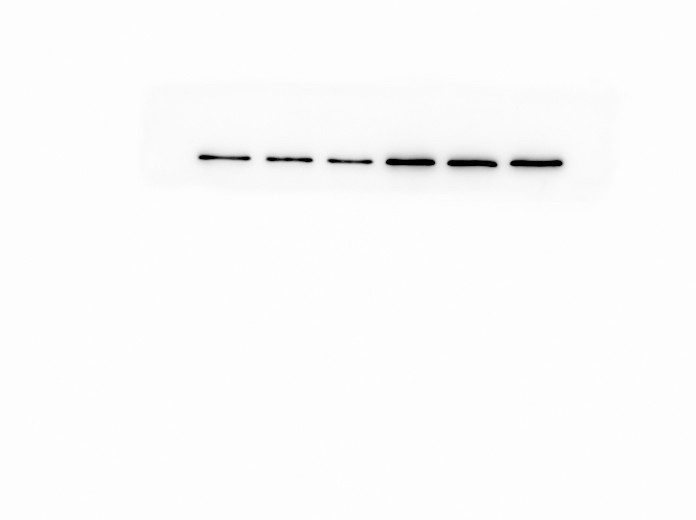


p-AKT 60 kDa

β-actin 54 kDa

l. figure4-p-AKT


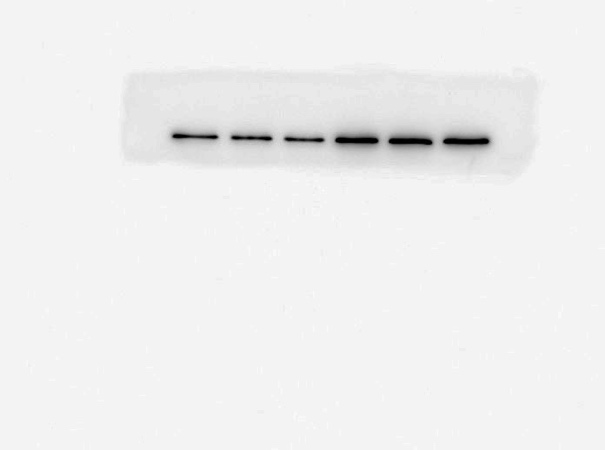


p-AKT 60 kDa

m. figure4-AKT n. figure4-β-actin


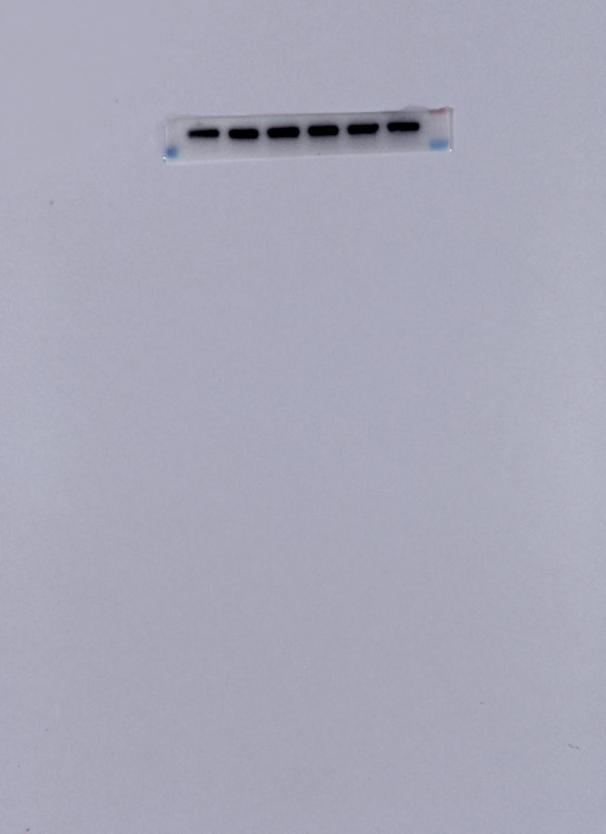

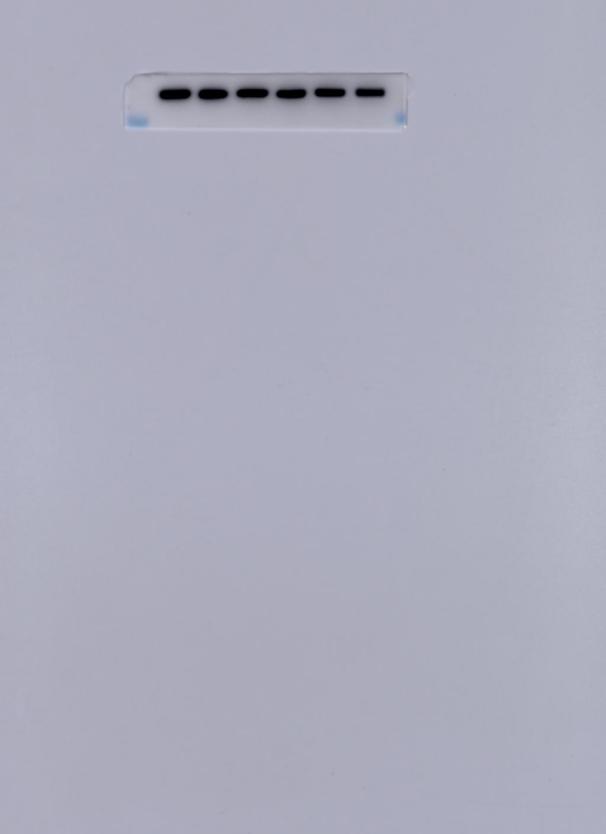


β-actin 45 kDa

AKT 60 kDa

o. figure6- AceCS1 p. figure6- ACSL1


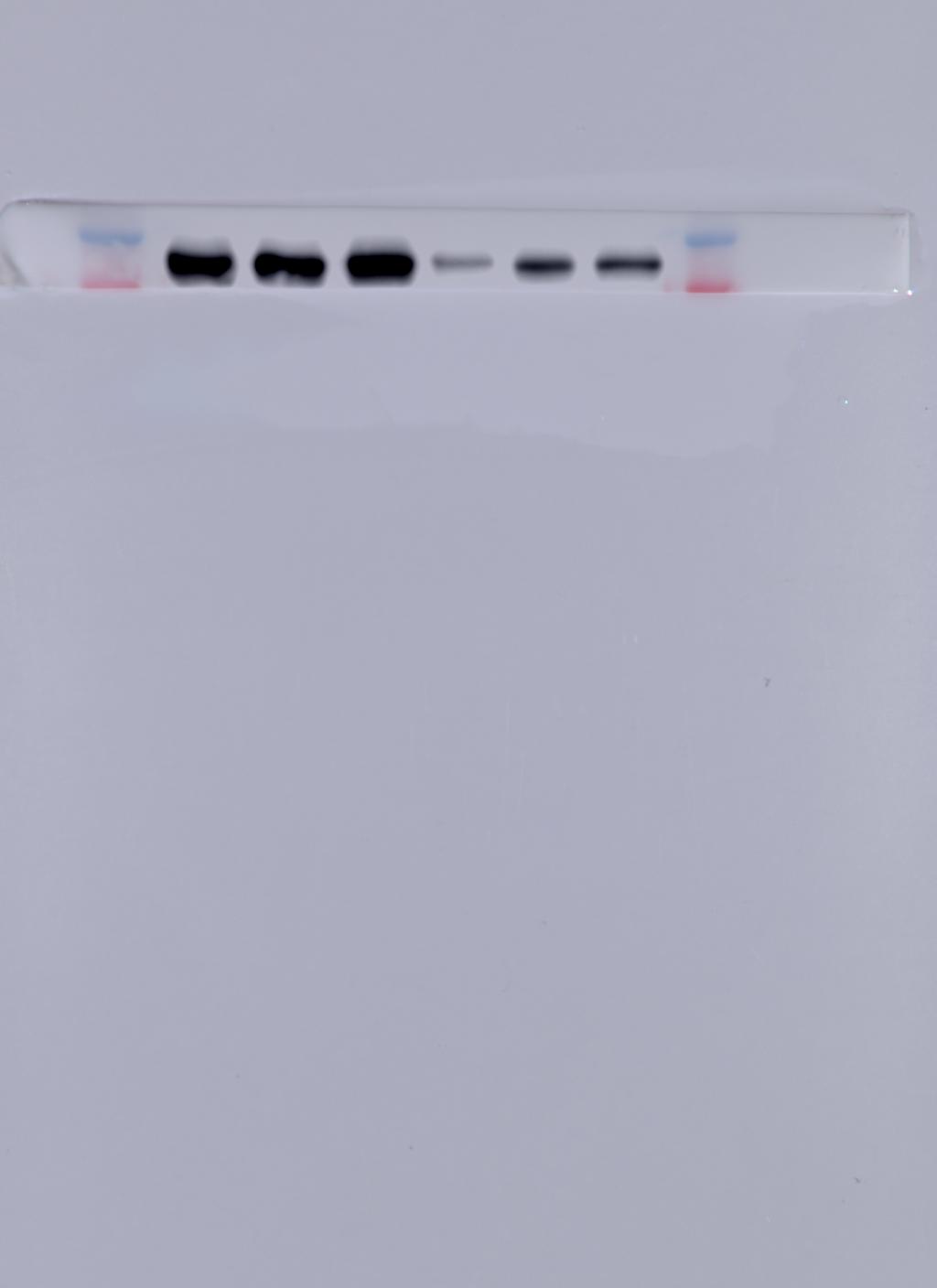

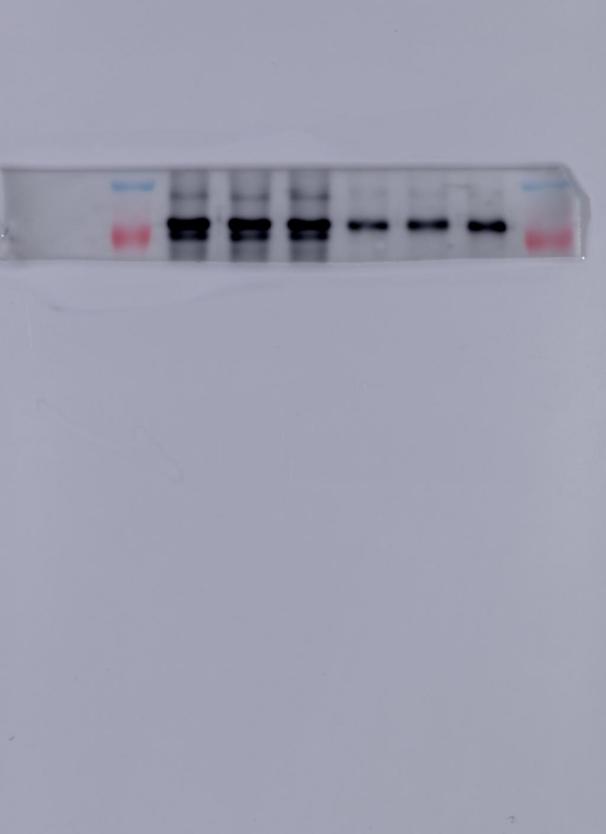


ACSL1 78 kDa

AceCS1 78 kDa

q. figure6- FAS r. figure6-p-ACC


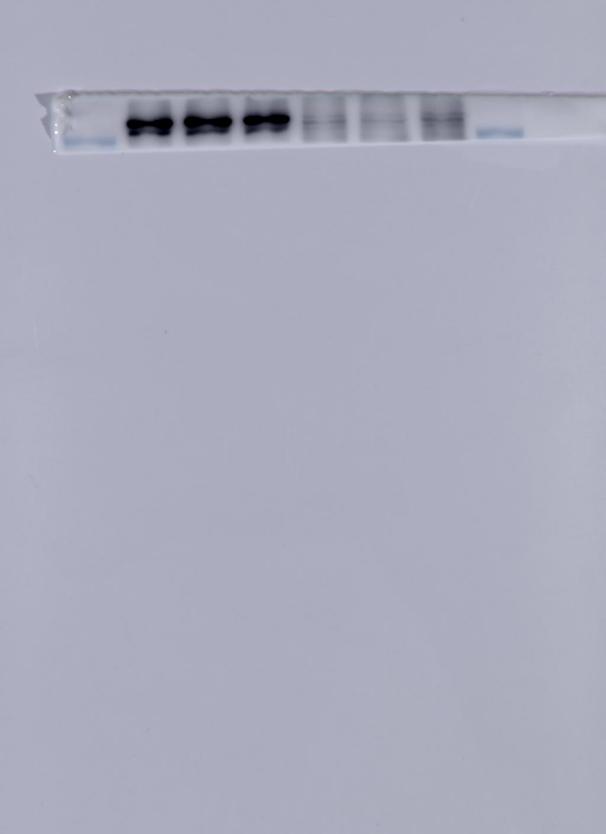

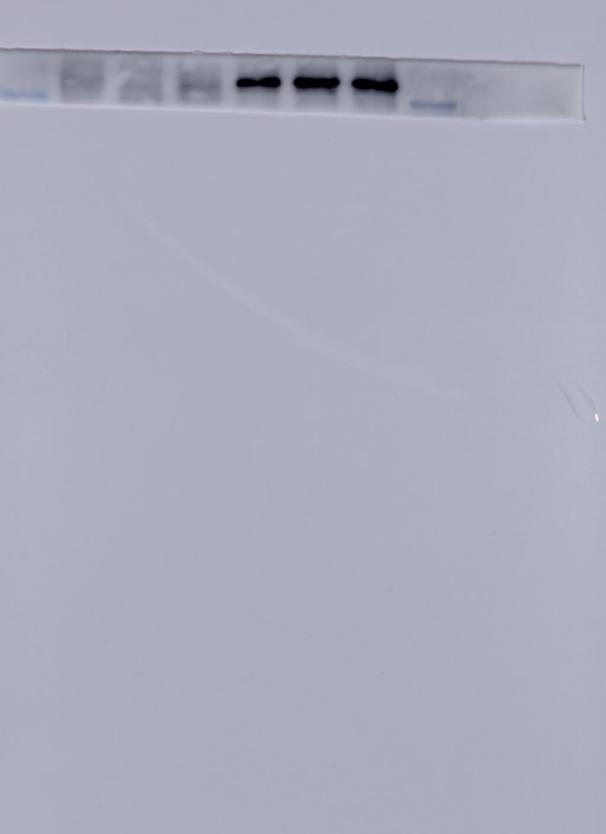


p-ACC 280 kDa

FAS 273 kDa

s. figure6-ADRP t. figure6-β-actin


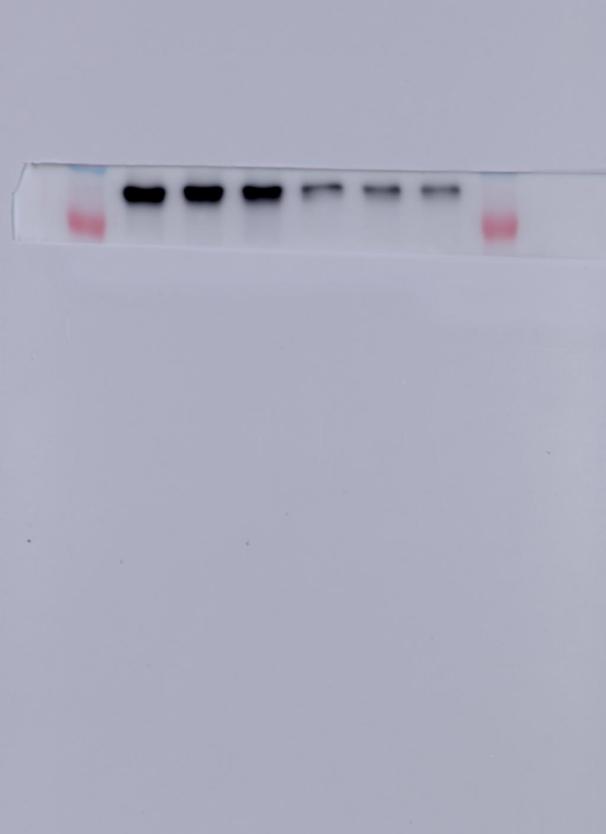

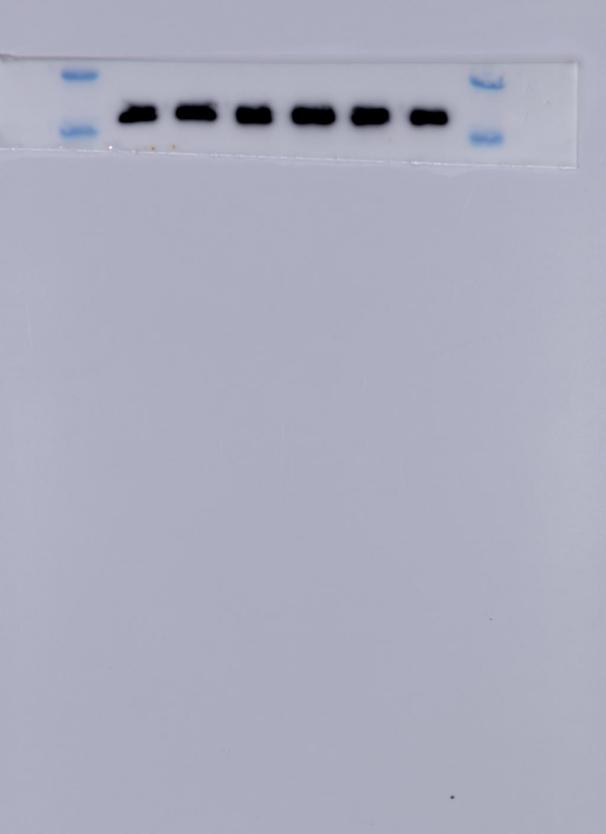


β-actin 45 kDa

ADRP 52 kDa

u. figure7A-PDH v. figure7A-OXPHOS
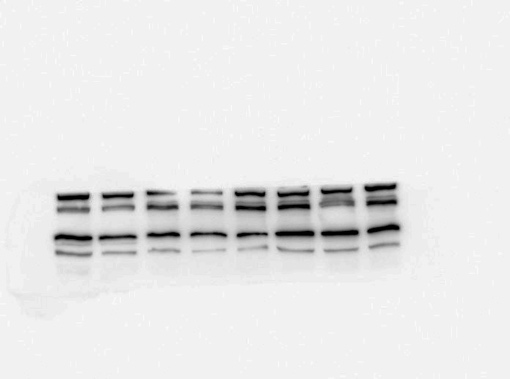

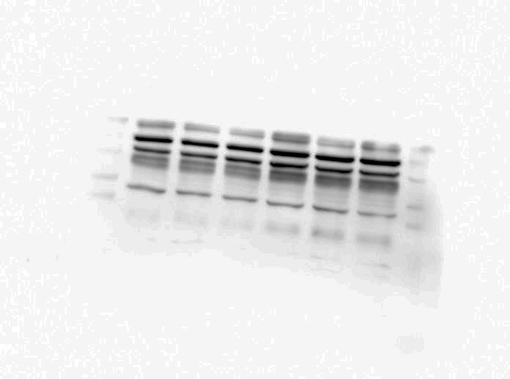


OXPHOS

20 kDa

30 kDa

40 kDa

48 kDa

55 kDa

PDH 43 kDa

w. figure7A-β-actin x. figure7C- AceCS1


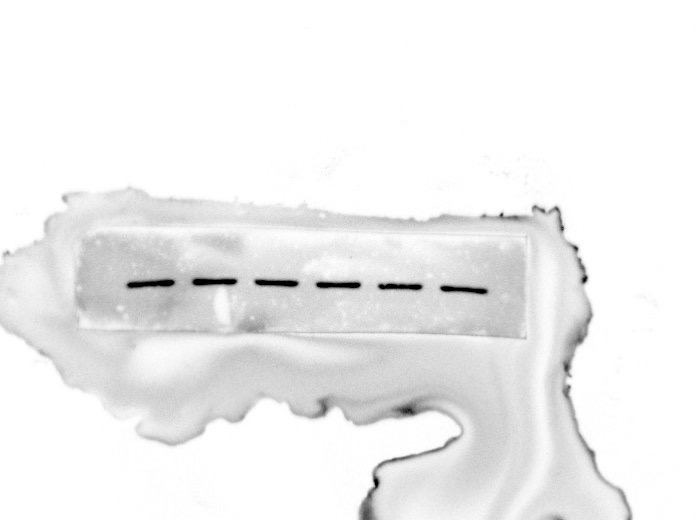

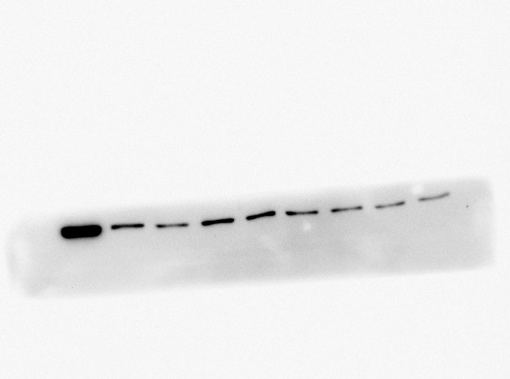


AceCS1 78 kDa

β-actin 43 kDa

y. figure7C- ACSL1 y. figure7C- ACSL1


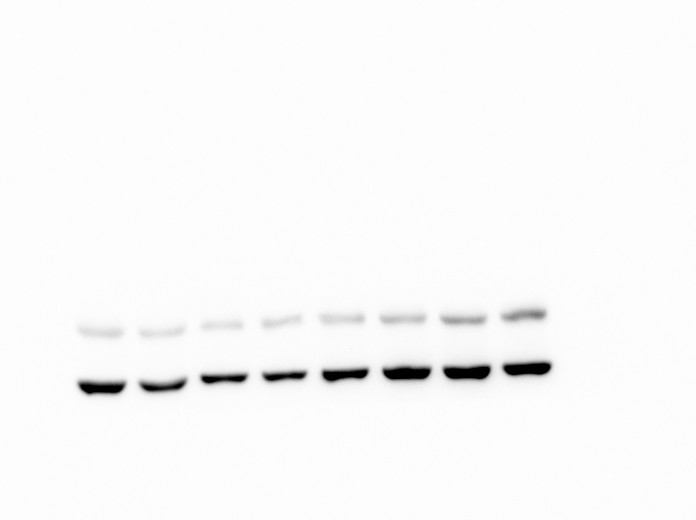

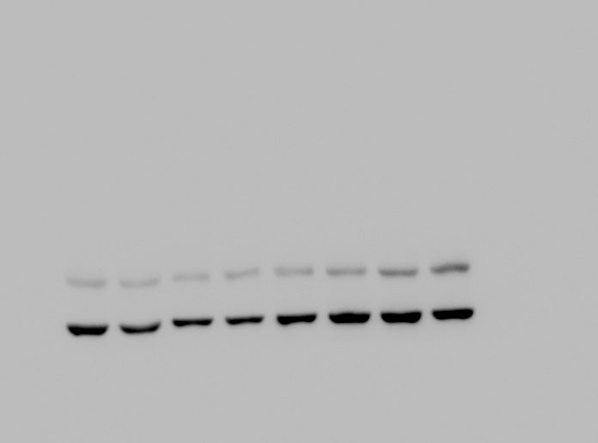


ACSL1 78 kDa

ACSL1 78 kDa

z. figure7C-FAS z. figure7C-FAS


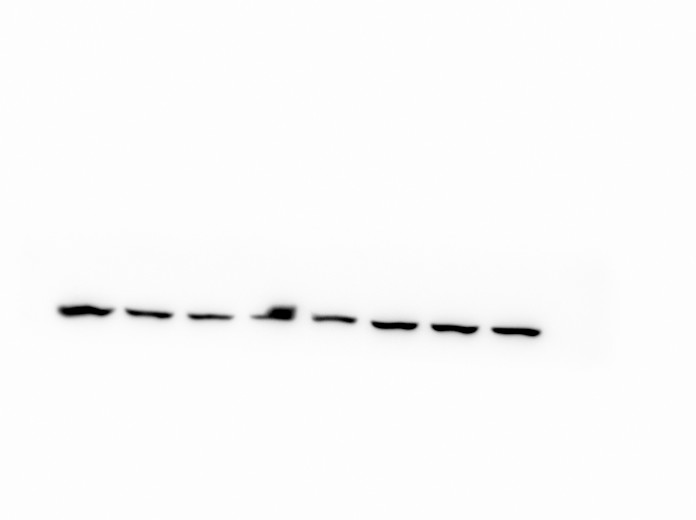

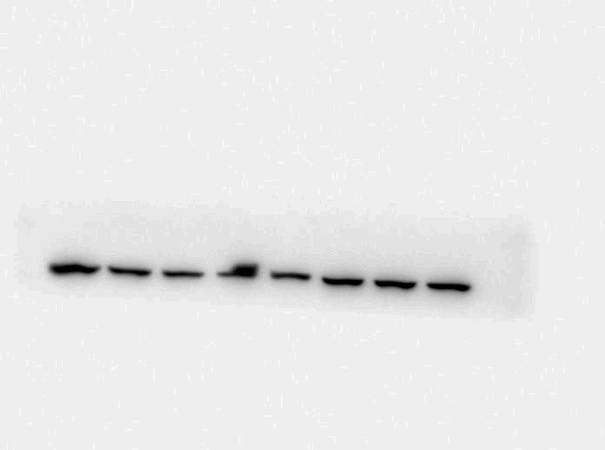


FAS 273 kDa

FAS 273 kDa

aa. figure7C-ADRP ab. Figure7C-β-actin


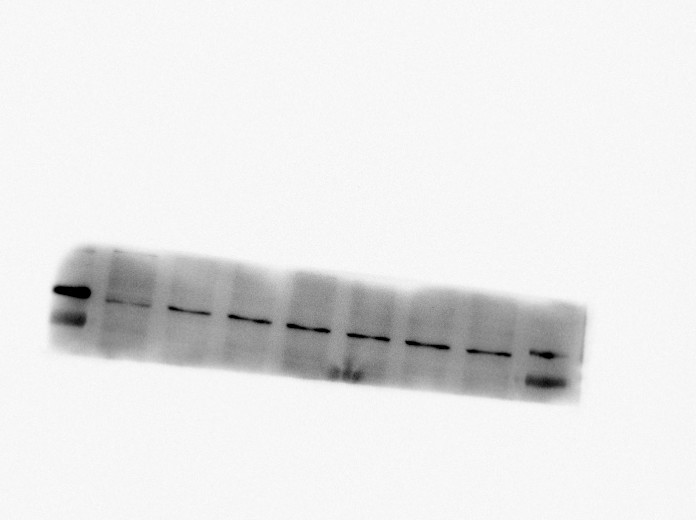

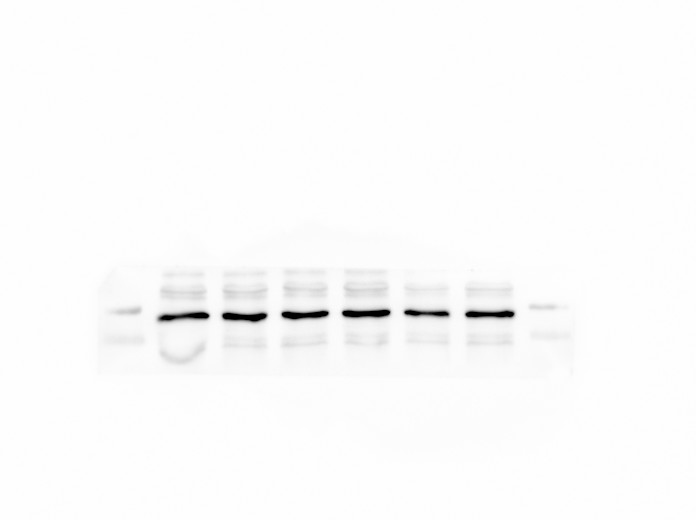


β-actin 45 kDa

ADRP 52 kDa

ab. Figure7C-β-actin


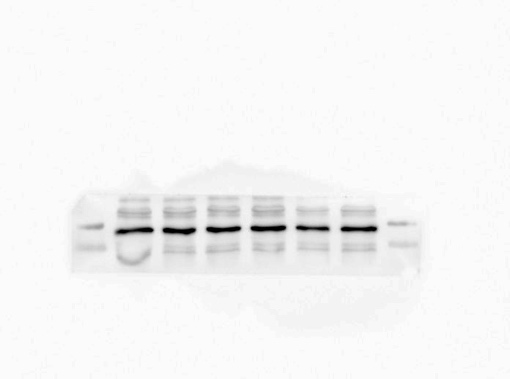


β-actin 45 kDa

**Supplementary Figure 2 original, unprocessed blots**

The blots are cut to the appropriate size prior to hybridisation with antibodies.
